# Salicylic Acid, Jasmonate, and Ethylene Contribute to Rice Defense Against White Tip Nematodes *Aphelenchoides besseyi*

**DOI:** 10.3389/fpls.2021.755802

**Published:** 2022-01-20

**Authors:** Jialian Xie, Fang Yang, Xing Xu, Yunliang Peng, Hongli Ji

**Affiliations:** MOA Key Laboratory of Integrated Management of Pests on Crops in Southwest China, Institute of Plant Protection, Sichuan Academy of Agricultural Sciences, Chengdu, China

**Keywords:** plant hormones, rice, Tetep, Nipponbare, rice white tip nematode

## Abstract

Plant hormones have a prominent place in the plant immune and defense mechanism. To gain more information about the plant hormone pathways involved in rice defense against nematodes, here, we studied the roles of three core hormones, namely, salicylic acid (SA), jasmonate (JA), and ethylene (ET) in rice defense to *Aphelenchoides besseyi* by using the susceptible variety, Nipponbare as well as the resistant variety Tetep. The data showed that Tetep exhibited pre- and post-invasion with suppression of nematode infection, development, and reproduction. The quantitative real-time (qRT)-PCR analysis of plant hormone marker genes in the two cultivars clearly revealed that all the SA-related genes were downregulated in susceptible Nipponbare plants but were significantly upregulated in resistant Tetep plants at the flowering stage. The exogenous application of the SA analog, benzo-1,2,3-thiadiazole-7-carbothioic acid *S*-methyl ester (BTH), methyl jasmonate (MeJA), and ethephon did induce rice resistance to *A. besseyi*, and the rice plants treated by hormone inhibitors increased susceptibility to *A. besseyi*. Similarly, corresponding transgenic biosynthesis or signaling mutants of those hormones also showed an increased susceptibility. Collectively, these results suggest that SA, JA, and ET play important defense roles in rice against *A. besseyi*.

## Introduction

Rice (*Oryza sativa*) is a major food source that feeds more than one-third of the world population. During the growth of rice plants, they are potential targets for more than 100 species of plant parasitic nematodes (Fortuner and Merny, 1979); *Aphelenchoides besseyi*, known as the rice white tip nematode, is one of the most important parasitic nematodes of rice (Duncan and Moens, 2013). As a seed-borne nematode, *A. besseyi* can survive on stored grain in anhydrobiosis for several years (Tiwari and Khare, 2003). After sowing, anabiotic *A. besseyi* rapidly become active and are attracted to rice meristematic areas. The nematodes migrate in the leaf or young tissue of rice as ecto- and endoparasites and cause a typical symptom of whitening and withering at the leaf tips (Togashi and Hoshino, 2001; Duncan and Moens, 2013). *A. besseyi* can be found in low numbers at all green tissues during early growth stages of rice; later, the nematodes enter spikelets before anthesis, and a rapid increase in the number of nematodes takes place at the flowering stage (Goto and Fukatsu, 1952). Around 30–70% reduction in yield caused by *A. besseyi* has been reported (Tikhonova, 1966; Muthukrishnan et al., 1974; Lin et al., 2004; Tulek and Cobanoglu, 2010); therefore, *A. besseyi* was listed in the top 10 plant-parasitic nematodes (Jones et al., 2013). To control this nematode, resistant cultivars offer the most favorable and durable options (De Waele, 2002), as has been proven in the United States (Prot and Rahman, 1994). In China, *A. besseyi* was first found in the 1940s and widely spread throughout the rice-growing regions (Pei et al., 2010; Wang et al., 2017); it causes 30–50% yield losses in flooded rice (Wang et al., 2014). Notably, 27 rice cultivars have been assessed to determine the resistance against *A. besseyi* in the field, it was found that 7 cultivars were resistant, and Tetep (i.e., an indica rice) showed high resistance to *A. besseyi* (Feng et al., 2014).

To minimize the damage caused by pathogens, plants have evolved an arsenal of immune and defense mechanisms, in which plant hormones play important signaling roles. Salicylic acid (SA), jasmonate (JA), and ethylene (ET) are the three classical hormones that generate and transmit signals to help host plants fight against pathogens (Grant and Jones, 2009; Robert-Seilaniantz et al., 2011). SA is usually activated when plants encounter threats from biotrophic and hemi-biotrophic pathogens, while JA and ET play key roles in the defense against necrotrophic pathogens, insects, and root-knot nematodes (Glazebrook, 2005; Howe and Jander, 2008; Nahar et al., 2011). During defense signaling, these three hormones can act synergistically or antagonistically (Koornneef and Pieterse, 2008; Pieterse et al., 2009). In general, JA and ET signaling pathways are believed to be synergistical in dicotyledon plants, while SA and JA/ET defense pathways are often found mutually antagonistic, but synergistic interactions between these two hormones have also been reported (Rojo et al., 2003; Lorenzo and Solano, 2005; Beckers and Spoel, 2006; Van Loon et al., 2006). Among others, SA, JA, or ET accumulation leads to the expression of pathogenesis-related (PR) genes such as *PR1*, *PR2*, and *PR3* in various tissues (López et al., 2008; Bari and Jones, 2009).

Recently, some studies have reported on the roles of SA, JA, and ET in rice defense toward plant-parasitic nematodes. For instance, it has been reported that JA pathway plays a crucial role in rice defense against the root-knot nematode *Meloidogyne graminicola*, while SA, JA, and ET biosynthesis pathways are equally important for defense against the migratory root nematode *Hirschmanniella oryzae* and rice stem nematode *Ditylenchus angustus* (Kyndt et al., 2012; Nahar et al., 2012; Khanam et al., 2018). There is little knowledge about the role of plant hormones in the interaction of rice plants and *A. besseyi*, which has a different strategy of multiplication and dispersal to root nematodes such as root-knot nematode or cyst nematode and rice stem nematode *D. angustus*.

To gain more information about the roles of plant hormone pathways involved in rice defense against *A. besseyi*, we first characterized the reaction of two rice cultivars upon *A. besseyi* infection, and then further clarified the role of SA, JA, or ET in the interaction of rice and *A. besseyi* by several approaches.

## Materials and Methods

### Plant Materials

The *NahG* line was a gift from Prof. Dr. Cheng-Cai Chu (Chinese Academy of Sciences, Beijing, China). The *Coi1-13* line was provided by Prof. Dr. Dong-Lei Yang (Nanjing Agricultural University, Nanjing, China). The *OsEin2b-RNAi* line was provided by Yi-Nong Yang (Pennsylvania State University, United States), and the *OsWRKY45-RNAi* transgenic line was a gift from H. Takatsuji (Plant Disease Resistance Research Unit, National Institute of Agrobiological Sciences, Ibaraki, Japan); all the above four lines were all with a background of Nipponbare. The mutant *hebiba* and its corresponding wild-type cv. Nihonmasari were generously provided by P. Nick (Karlsruhe University, Karlsruhe, German). Cultivar Tetep and Nipponbare were maintained in the lab, which were initially obtained from rice breeders.

Rice cultivar Tetep and Nipponbare were used to detect the level of resistance/susceptibility to *A. besseyi* and analyze the expression of plant hormone-related genes. Rice cultivar Nipponbare was also used to be sprayed by hormones or hormone inhibitors to clarify the roles of hormones in nematode defense. Five rice mutants or transgenic lines including an SA-deficient transgenic *NahG* line, a JA response-deficient mutant line *Coi1-13*, a transgenic line *OsEin2b-RNAi*, which is with a silenced expression of ET signaling gene *OsEin2b*, an *OsWRKY45-RNAi* transgenic line, and a JA biosynthesis mutant *hebiba* were used to further obtain a detailed role of the three hormone pathways in the interaction of rice and *A. besseyi*.

### Growth Conditions

All seeds used in this research were first soaked in hot water at 56°C for 15 min to kill the seed-borne nematodes (Fortuner and Orton Williams, 1975), then germinated on wet filter paper for 3 days at 28°C, subsequently transferred to autoclaved sand mixed with 0.2% (m/v) superabsorbent polymer (SAP) substrate (Reversat et al., 1999) in 10 ml centrifuge tubes, and further were grown at 26°C under a 16/8 h light regime. To harvest the rice grains and further count the nematodes, the seedlings were transplanted into a greenhouse plot at 25 days after inoculation (dai) with a density of 250 mixed infective stages of *A. besseyi* per plant and grown at approximately 28–30°C and 60–70% relative humidity (RH). Plots were 1.5 m × 2.0 m and caged with nylon nets to protect against insects. An earth levee of 25 cm height was made around each plot to maintain the water level and to isolate each plot.

### Nematode Culture and Inoculation

*Aphelenchoides besseyi* was isolated from infected rice seeds of AnHui1 (*O. sativa* subsp. *japonica*) by soaking seeds in tap water for 72 h. Then, nematodes were collected and surface-sterilized with 0.1% streptomycin sulfate for 10 min, washed three times with sterile double-distilled water, and subsequently cultured on carrot discs at 25°C for 30 days as described in the study by Tulek et al. (2009).

The nematodes were then collected in tap water, and approximately 250 nematodes were inoculated to each plant by using water flotation method described by Xie et al. (2019). Briefly, hot water-treated seeds were germinated for 3 days at 28°C and planted individually into the center of a 10 ml centrifuge tube containing 4 ml SAP substrate; the tubes were capped and the plants were grown for 3 days at 26°C under a 16 h/8 h light regime. Subsequently, 2 ml of nematode suspensions in tap water containing 250 mixed infective stages of nematodes were added to each plant to submerge the growth site of the seedling. The tubes were capped for another 5 days to retain the moisture. Clean tap water without nematodes was used as the mock control.

### Chemical Treatment

The hormone analogs benzo-1,2,3-thiadiazole-7-carbothioic acid *S*-methyl ester (BTH), methyl jasmonate (MeJA), and ethephon were purchased from Sigma-Aldrich (Sigma, St. Louis, United States). Diethyldithiocarbamic acid [DIECA (Farmer et al., 1994)], a JA biosynthesis inhibitor; aminooxyacetic acid [AOA (Iwai et al., 2006; Mao et al., 2006)], an inhibitor of ET biosynthesis, and L-2-aminooxy-3-phenylpropionic acid [AOPP (Amrhein and Gödeke, 1977)], an inhibitor of phenylalanine ammonia-lyase (PAL) activity were bought from FUJIFILM Wako Pure Chemical Corporation (Neuss, Deutschland) and Sigma-Aldrich (Sigma, St. Louis, United States). Two concentrations of hormones or analogs were used in the study, i.e., MeJA (250 and 500 μM), BTH (125 and 250 μM), and ethephon (250 and 500 μM). The following concentrations of the hormone inhibitor were used in this study: DIECA (100 μM), AOA (20 mM), and AOPP (100 μM). All chemicals were dissolved in water containing 0.02% (v/v) Tween 20.

The chemical treatment was examined at two stages in Nipponbare. For the analysis of the effects of chemicals on nematode infection at the seedling stage, the prepared chemicals or water control containing 0.02% (v/v) Tween 20 were sprayed with a perfume sprayer onto the leaves of six-day-old seedlings; the nematode of mixed infective stages with a density of 250 per plant were inoculated at 24 h after spraying, and a number of nematodes were collected from above ground parts at 25 dai. For the analysis of the effects of chemicals in nematode infection at flowering stage, 250 of mixed infective stages nematodes were inoculated individually onto seven-day-old seedlings and then transplanted into a greenhouse plot at 25 dai for further growth. The prepared chemicals or water control containing 0.02% (v/v) Tween 20 were sprayed onto the leaves and panicles at day one of flowering, and the number of nematodes were collected from grains at the ripen stage.

### Nematode Counting

To analyze the nematode reproduction dynamics in Nipponbare and Tetep, nematodes were collected from the above ground parts at 15 dai, tillering stage (about 30 dai), jointing stage (about 45 dai), from panicles at booting stage (about 60 dai) and flowering stage (about 70 dai), and from grains at ripen stage (about 100 dai). To confirm the effects of plant hormones on nematode infection, nematodes on mutant lines or chemical-treated plants were collected at 25 dai and grains. The white-tip symptoms were observed and recorded at tillering stage. Symptom rates (%) = The number of plants showing white-tip symptoms/the number of inoculated plants × 100%.

All the counts in this study were performed according to Xie et al. (2019). Briefly, the above ground part of seedlings or panicles was cut into approximately 1 cm long pieces, immersed in 0.01% Tween 20, placed on a shaker at 40 rpm for 24 h, and then, the water was collected and the materials were rinsed twice; all the water was collected into a counting dish, and the nematodes in water were counted under a stereomicroscope. Each experiment or treatment had three biological replicates, and each replicate contained 15 plants. For evaluation of the nematode population in grains, a total of randomly selected 100 mixed seeds from one replicate of one treatment were collected and were gently ground to separate glumes and seeds, and then the glumes and grains were soaked in tap water in a Petri dish on a shaker at 40 rpm for 8 h and washed twice. Nematode observation was performed the same as described above under a stereomicroscope. Each replicate contains five randomly mixed 100 grains; three independent biological replicates were recorded.

### RNA Extraction, cDNA Synthesis, and Quantitative Real-Time-PCR

For quantitative real-time (qRT)-PCR, the above-ground tissues (including all the leaves and stem) of Tetep and Nipponbare were separately collected at 3, 5, 7, and 14 dai, and the panicles were collected at the flowering stage after nematode inoculation. The tissues of clean tap water inoculation were served as the corresponding control. RNA was extracted using the TRIzol reagent (Invitrogen, Breda, Netherlands), and the extracted RNA was treated with DNaseI (Thermo Fisher Scientific) to remove all contaminating DNA as follows: added 1 μl (1 U) DNase I, 1 μg extracted RNA, 1 μl of 10× reaction buffer with 25 mM MgCl_2_, and DEPC-treated water into RNase-free tube to a final volume of 10 μl and then incubated at 37°C for 30 min; later, 1 μl 50 mM EDTA was added and incubated at 65°C for 10 min. The RNA concentration and purity were measured using a NanoDrop 2000 (Thermo Scientific). The first-strand cDNA synthesis was generated using RevertAid First Strand cDNA Synthesis Kit (Thermo Fisher Scientific, Lithuania). The PowerUp™ SYBR™ Green Master Mix (Thermo Fisher Scientific, Lithuania) was used to perform qRT-PCR: Green Master Mix (2×) 10 μl, forward primer 1 μl (10 ng/μl), reverse primer 1 μl (10 ng/μl), cDNA 1 μl (20 ng/μl), and nuclease-free water 7 μl. qRT-PCR was performed under the following conditions: 5 min at 95°C and 45 cycles of (10 s at 95°C and 30 s at 59°C). After the PCR reaction, a melting curve was generated by gradually increasing the temperature to 95°C to test for amplicon specificity. All reactions were performed in three technical replicates on Bio-Rad CFX connect (Bio-Rad, United States) and analyzed using R version 3.3.0 (R Core Team, 2016). Each biological replicate composed of a pool of eight individual plants or panicles. The experiment was independently repeated three times. Expression data were normalized using two reference genes. Primer pairs for each target or reference gene are listed in Table 1.

**TABLE 1:** Primer pairs for each target or reference gene in quantitative real-time (qRT)-PCR.

Target gene	Forward primer (5′–3′)	Reverse primer (5′–3′)	Rice genome locus number
*OsPAL*	TGTGCGTGCTTCTGCTGCTG	AGGGTGTTGATGCGCACGAG	LOC_Os02g41630
*OsICS1*	TGTCCCCACAAAGGCATCCTGG	TGGCCCTCAACCTTTAAACATGCC	LOC_Os09g19734
*OsWRKY45*	AATTCGGTGGTCGTCAAGAA	AAGTAGGCCTTTGGGTGCTT	LOC_Os05g0323900
*OsNPR1*	CACGCCTAAGCCTCGGATTA	TCAGTGAGCAGCATCCTGACT	LOC_Os01g0194300
*OsAOS2*	CAATACGTGTACTGGTCGAATGG	AAGGTGTCGTACCGGAGGAA	LOC_Os03g12500
*OsLOX3*	TCGCATTGGCTCGCACCCT	TCGTCTTCTTCAGCCGCACGAT	LOC_Os03g0700400
*OsJMT1*	CACGGTCAGTCCAAAGATGA	CTCAACCGTTTTGGCAAACT	LOC_Os06g0314600
*OsJAmyb*	GAGGACCAGAGTGCAAAAGC	CATGGCATCCTTGAACCTCT	LOC_Os11g45740
*OsACO7*	GGACTACTACCAGGGCACCA	GATTAGCGCACGCGATTTTA	LOC_Os01g39860
*OsACS1*	GATGGTCTCGGATGATCACA	GTCGGGGGAAAACTGAAAAT	LOC_Os03g51740
*OsEIN2a*	TAGGGGGACTTTGACCATTG	TGGAAGGGACCAGAAGTGTT	LOC_Os07g06130
*OsERF1*	AAGGGTCATAATTCGCGTCA	TCCACACCACAAGACATCGT	LOC_Os04g46220
*OsPR1a*	TCGTATGCTATGCTACGTGTTT	CACTAAGCAAATACGGCTGACA	LOC_Os07g0418500
*OsPR1b*	GGCAACTTCGTCGGACAGA	CCGTGGACCTGTTTACATTTT	LOC_Os01g28450
*OsPR10*	CCCTGCCGAATACGCCTAA	CTCAAACGCCACGAGAATTTG	LOC_Os12g36880
*OsEXP*	TGTGAGCAGCTTCTCGTTTG	TGTTGTTGCCTGTGAGATCG	LOC_Os03g27010
*OsEXPnarsai*	CACGTTACGGTGACACCTTTT	GACGCTCTCCTTCTTCCTCAG	LOC_Os07g02340.1

### Data Analysis

The data were analyzed using the R version 3.3.0 software. Normality and equality of variances were tested with Shapiro–Wilk normality test (α = 0.05) and Bartlett test (α = 0.05), respectively. Normally distributed data were analyzed using an independent samples *t*-test or ANOVA followed by individual comparisons with the Tukey test (α = 0.05). Non-parametric testing was carried out with the Kruskal–Wallis test with α = 0.05. All data except qRT-PCR in the same treatment were not significantly different between replicates in this research; therefore, the data presented in all the results are the means of three replicates.

## Results

### The Reproduction Dynamics of *Aphelenchoides besseyi* in Nipponbare and Tetep

**FIGURE 1:**
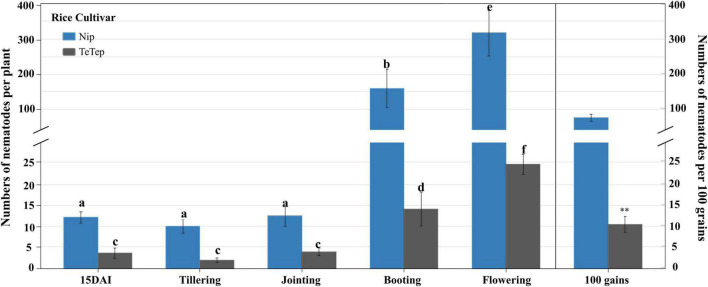
Number of *Aphelenchoides besseyi* of two rice cultivars Nipponbare and Tetep at different stages. Six-day-old rice plants were inoculated with 250 mixed stages of *A. besseyi*. The number of nematodes was observed at 15 dai, tillering, jointing, booting, flowering stages, and mature grains. Bars in the left part are represented as the means ± SE from 3*n* = 45 plants. Bars in the right part were represented as the means ± SE from 3*n* = 15 in 100 grains. Different letters indicate statistically significant differences (Tukey test with α = 0.05). The symbol “^**^” indicates statistically significant differences with *t*-test at α = 0.01.

The number of nematodes per plant in Nipponbare was significantly higher than the ones in Tetep at all the stages tested (Figure 1). There were no significant differences between the number of nematodes per plant at 15 dai, tillering stage, and jointing stage either in Nipponbare or in Tetep. However, a 13- to 26-fold increase of nematode numbers was observed in Nipponbare at booting and flowering stages, and the number reached 160.0 and 310.1 per plant, respectively. The number of nematodes only slightly increased in Tetep at these two stages, with a four- to eightfold increase up to 14.2 and 24.8 nematodes per plant. Besides, there were, on average, 10.6 nematodes in 100 grains for Tetep, which was significantly lower than the average of 92.7 nematodes in 100 grains for Nipponbare.

The typical white tip symptom is the production of chlorotic tips on flag leaves starting at tillering, which may subsequently necrotize. This symptom was recorded in both Nipponbare and Tetep at the tillering stage, at a rate of 72.9% for Nipponbare, while in Tetep, no typical symptom was observed at the whole growth stages (Supplementary Figure 1).

### The Expression of Plant Hormone Marker Genes at Seedling Stages in Rice Plants Under Attack by *Aphelenchoides besseyi*

**FIGURE 2:**
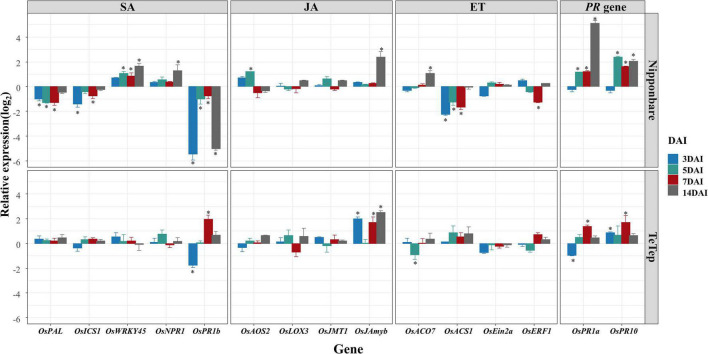
Quantitative real-time (qRT)-PCR analysis on the selected defense-related genes in shoot tissues in two cultivars at seedling stage. Bars represent mean expression levels and SE from four technical replicates of one biological replicate, containing a pool of eight plants. The other two biological replicates show similar results. Data are shown as relative expression levels of infected tissues in comparison with the control tissue (uninfected tissue). Gene expression levels were normalized using two internal reference genes, *OsEXP* and *OsEXPNarsai*. Asterisks indicate statistically significant (α = 0.05) differential expression in comparison with control plants, performed using R version 3.3.0.

The possible roles of plant hormones during the interactions of rice and *A. besseyi* were investigated by evaluating the expression levels of 13 selected SA, JA, or ET marker genes and 2 PR genes in Nipponbare and Tetep at early seedling stages (Figure 2).

For SA signaling pathway, two biosynthesis genes *OsICS1* and *OsPAL1*, two SA response genes *OsWRKY45* and *OsNPR1*, and one SA-inducible PR gene *OsPR1b* were analyzed. During the interaction of Nipponbare and *A. besseyi*, the expression levels of *OsPAL1* and *OsICS1* were consistently downregulated at all time points compared with those in mock control. However, the transcript level of *OsWRKY45* was slightly increased in shoot tissues at 3 dai but was significantly upregulated at 5, 7, and 14 dai. The expression of *OsNPR1* was not significantly affected by *A. besseyi* at the most of stages; however, the transcription level of *OsNPR1* was significantly upregulated at 14 dai. The *OsPR1b* expression levels were downregulated at all times, especially at 3 and 14 dai. During the interaction of Tetep and *A. besseyi*, *OsICS1*, *OsPAL1*, *OsWRKY45*, and *OsNPR1* showed no significant changes upon *A. besseyi* inoculation at all time points, while downregulation at 3 dai and upregulation at 7 and 14 dai were observed in *OsPR1b*.

To investigate the JA response, *OsAOS2* (a key enzyme in JA biosynthesis), *OsLOX3* (a JA synthesis pathway-related gene), *OsJMT1* (an enzyme that converts JA to the volatile component MeJA), and *OsJAmyb* (a JA-inducible Myb transcription factor) were analyzed. The expression levels of *OsAOS2* at 5 dai and *OsJAmyb* at 14 dai were significantly upregulated in Nipponbare, while the other genes had no significant changes at all time points. In contrast, during the interaction of Tetep and *A. besseyi*, the expression of *OsJAmyb* was significantly upregulated at 3, 7, and 14 dai, while all the other genes showed similar expression levels compared with control plants.

Genes such as *OsACO7*, *OsACS1*, *OsEin2b*, and *OsERF1* that play key roles in ET biosynthesis or signaling were analyzed by using qRT-PCR. The expression of *OsACO7* was significantly upregulated at 14 dai in Nipponbare but was downregulated at 5 dai in Tetep. The expression of *OsACS1* was significantly downregulated in Nipponbare at 3, 5, and 7 dai, but only minor upregulation was detected in Tetep at the same stages. No significant differences were detected for *OsEin2b* between the inoculated and control plants. Nevertheless, ET-inducible gene *OsERF1* appeared to be significantly down-regulated at 7 dai in Nipponbare, but no significant transcription alteration was detected in Tetep at these stages.

To assess the general defense responses in these two cultivars, after *A. besseyi* inoculation, the expressions of two PR genes *OsPR1a* and *OsPR10* were analyzed. *OsPR1a* and *OsPR10* were both significantly upregulated at all the time points except at 3 dai in Nipponbare. In contrast, the mRNA level of *OsPR1a* was dramatically downregulated at 3 dai and upregulated at 7 dai in Tetep; the expression levels of *OsPR10* were significantly induced at 3 and 7 dai.

### The Expression of Plant Hormone Marker Genes at Flowering Stage During the Infection of *Aphelenchoides besseyi*

**FIGURE 3:**
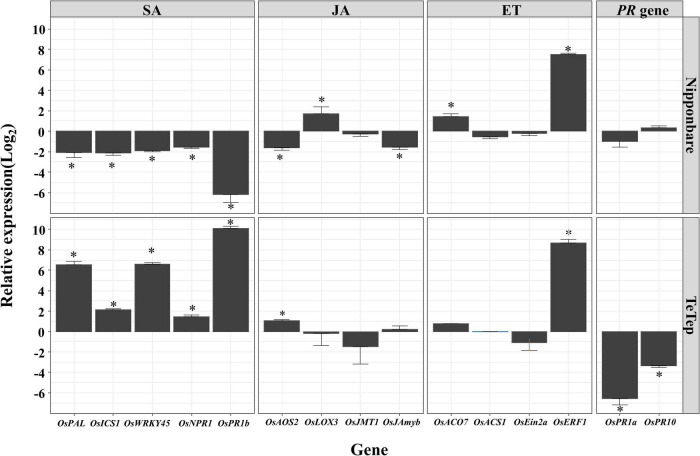
Quantitative real-time (qRT)-PCR analysis on the selected defense-related genes in panicle tissues in two cultivars at flowering stage. Bars represent mean expression levels and SE from four technical replicates of one biological replicate, containing a pool of eight panicles. The other two biological replicates show similar results. Data are shown as relative expression levels of infected tissues in comparison with the control tissue (uninfected tissue). Gene expression levels were normalized using two internal reference genes, *OsEXP* and *OsEXPNarsai*. Asterisks indicate statistically significant (α = 0.05) differential expression in comparison with control plants, performed using R version 3.3.0.

As shown in Figure 1, the number of nematodes is massively increasing at the flowering stage in rice panicles. Therefore, the expression levels of the same plant hormone marker genes in panicles were analyzed to investigate the role of plant hormones during *A. besseyi* infection at the flowering stage (Figure 3).

During the interaction of Nipponbare and *A. besseyi*, the SA marker genes were significantly downregulated at this stage compared with corresponding control. On the contrary, all these genes showed significantly upregulated expression levels in Tetep upon *A. besseyi* infection at this time points compared with those in the control.

For the key genes involved in JA response, the expressions of *OsAOS2* and *OsJAmyb* were down-regulated, while *OsLOX3* was upregulated at the flowering stage in Nipponbare. In contrast, in Tetep, only the expression of *OsAOS2* was upregulated by *A. besseyi* infection, and all the other genes showed similar expression level compared with the control.

For ET signaling marker genes, *OsERF1* appeared to be significantly upregulated both in Nipponbare and Tetep. *OsACO7* was significantly upregulated in Nipponbare, but this gene was not affected by *A. besseyi* infection in Tetep. Moreover, the other two genes showed no significant differences in the transcription level in both cultivars compared with the corresponding controls.

*OsPR1a* and *OsPR10* were not significantly affected in Nipponbare but were both significantly downregulated in Tetep at the flowering stage after nematode infection.

### The Effect of the Application of Hormones or Hormone Inhibitors on Nipponbare Susceptibility to *Aphelenchoides besseyi*

**FIGURE 4:**
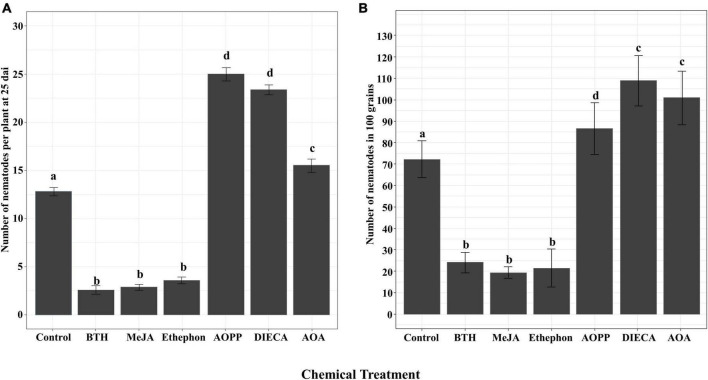
Effects of foliar application of plant hormones and hormone biosynthesis inhibitors on rice defense against *Aphelenchoides besseyi* infection. Shoots of six-day-old plants were sprayed until runoff with 125 μM BTH, 250 μM MeJA, 250 μM ethephon, 100 μM DIECA, 100 μM AOPP, and 20 mM AOA or water. At 24 h after treatment, plants were inoculated with 250 mixed stages of *A. besseyi*. **(A)** The number of nematodes per plant at 25 dai. Bars represent means ± SE from 3*n* = 45 plants. **(B)** The number of nematodes in 100 mature grains. Bars represent means ± SE from 3*n* = 15 in 100 grains. Different letters indicate statistically significant differences (Tukey test with α = 0.05). MeJA, methyl jasmonate; ethephon (converted to ET in the plant); BTH, benzathiadiazole (SA analog); AOA, aminooxyacetic acid (ET biosynthesis inhibitor); DIECA, diethyldithiocarbamic acid (JA biosynthesis inhibitor); AOPP, L-2-aminooxy-3-phenylpropionic acid (inhibitor of biosynthesis of phenylpropanoids, including SA).

To analyze the role of SA, JA, and ET in rice defense against *A. besseyi*, the number of nematodes was examined at two stages in Nipponbare. The results indicated that both in plants of 25 dai and in grains, the number of *A. besseyi* significantly reduced in hormone-treated plants when compared with the corresponding control in two concentrations (Figure 4 and Supplementary Figure 2), and the results showed the two concentrations had similar effects on nematode infection. As the results showed in Figure 4, in BTH-, MeJA-, and ethephon-treated plants, the number of nematodes exhibited decrease of 79.91, 77.58, and 71.47% at 25 dai, respectively. While in grains, BTH-, MeJA-, and ethephon-treated plants showed 63.71, 73.96, and 70.39% nematode reductions, respectively.

In contrast, chemical inhibition of the phenylpropanoid pathway with AOPP, JA biosynthesis with DIECA, or ET production with AOA all resulted in a significantly increased susceptibility toward *A. besseyi* both in 25 dai plants and grains (Figure 4). In 25 dai plants and grains, compared with those in control, AOPP-, DIECA-, and AOA-treated plants showed an increase of 91.51 and 19.76%, 83.84 and 50.78%, and 16.43 and 39.70% nematodes, respectively.

### Susceptibility of Rice Hormone Pathway Mutants to *Aphelenchoides besseyi*

**FIGURE 5:**
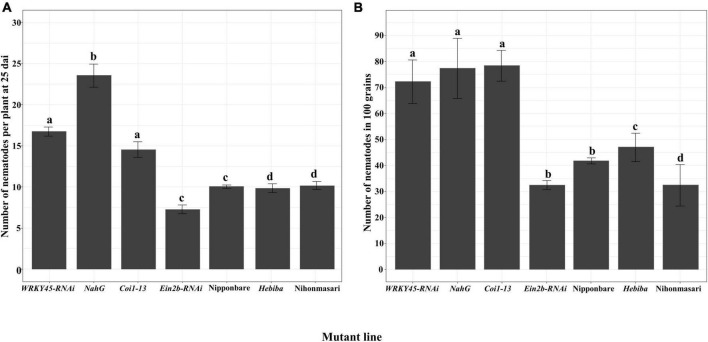
The number of *A. besseyi* on hormone biosynthesis or signaling deficient lines. Seven-day-old plants were inoculated with 250 mixed stages of *Aphelenchoides besseyi*. **(A)** The number of nematodes per plant at 25 dai. Bars represent means ± SE from 3*n* = 45 plants. **(B)** The number of nematodes in 100 mature grains. Bars represent means ± SE from 3*n* = 15 in 100 grains. Different letters indicate statistically significant differences (Tukey test with α = 0.05). *WRKY45-RNAi*, SA signaling deficient line; *NahG*, SA biosynthesis deficient line; *Ein2b-RNAi*, ET-insensitive line; *Coi1-13*, JA-insensitive line and their corresponding wild-type Nipponbare; *Hebiba*, JA biosynthesis mutant and its corresponding wild-type Nihonmasari.

To obtain a more detailed understanding of the role of these defense hormone pathways against *A. besseyi*, the number of nematodes was evaluated in the mutants or transgenic lines impaired in hormone pathways. Both in 25 dai plants and grains, the SA-signaling deficient mutant *WRKY45-RNAi*, the SA-deficient mutant *NahG*, and the JA insensitive mutant *Coi1-13* all showed a significant increase in the number of nematodes compared with the corresponding control of Nipponbare. Nevertheless, the ET-insensitive mutant *EIN2b-RNAi* did not have a significant change in the number of nematodes compared with that in control either in 25 dai plants or grains. The nematode number in the JA biosynthesis mutant *hebiba* had no significant change in 25 dai plants but was significantly increased in grains in comparison with control Nihonmasari (Figure 5).

## Discussion

It has been reported that Tetep is highly resistant to *A. besseyi* according to the few nematodes in 100 grains and the lack of “white tip” symptom, but the underlying mechanism remains largely unknown (Feng et al., 2014). To gain insights into the defense mechanisms of Tetep against *A. besseyi*, in this study, the number of nematodes and the expression levels of plant hormone marker genes were investigated in cultivar Tetep and Nipponbare. The number of nematodes in Tetep were significantly lower at all the time points as well as in 100 grains compared with Nipponbare, which indicates that the resistance in Tetep may be associated with the reduced numbers of invasion, development, and reproduction of *A. besseyi*. The same resistance mechanisms were also observed in previous studies on cultivars resistant to other nematodes (Linsell et al., 2014; Khanam et al., 2018; Lahari et al., 2020; Nzogela et al., 2020).

Several studies have been conducted to explore the role of SA, JA, and ET during the infection of parasitic nematodes in rice or dicotyledonous plants (Bhattarai et al., 2008; Uehara et al., 2010; Nahar et al., 2011, 2012; Khanam et al., 2018). Our study aims to provide a characterization of the role of SA, JA, or ET in rice defense against the foliar nematode *A. besseyi*, which has a different strategy of multiplication and dispersal compared with root nematodes or stem nematodes (Huang and Huang, 1972; Nandakumar et al., 1975).

The plant hormone SA plays an important role as a signaling molecule during the defense reaction on pathogen infection (Lefevere et al., 2020). SA is generally linked with defense against biotrophic pathogens and usually activated in systemic acquired resistance (SAR), which is also accompanied by the induction of a set of PR genes (Van Loon et al., 2006; Vlot et al., 2009). It is widely accepted that SA in plants is synthesized by both isochorismate synthase (ICS) and PAL, which starts from chorismate (Lefevere et al., 2020), and SA-signaling pathway in rice branches into *WRKY45*-dependent and *NPR1*-dependent sub-pathways (Shimono et al., 2007; Sugano et al., 2010). It has been found that foliar application of BTH to rice plants only resulted in a minor induction of systemic defense against the root-knot nematode *M. graminicola*; however, BTH could induce stronger defense in rice against migratory root nematode *H. oryzae* and rice stem nematode *D. angustus* (Nahar et al., 2012; Khanam et al., 2018) as well as other sedentary nematodes (Owen et al., 2002; Nandi et al., 2003; Branch et al., 2004; Wubben et al., 2008). Our expression analyses of SA biosynthesis in Nipponbare and Tetep showed that the related genes were downregulated in infected susceptible cultivar Nipponbare but were hardly affected in resistant cultivar Tetep at seedling stages. However, SA signaling genes such as *OsWRKY45* and *OsNPR1* were upregulated at early growth stage of rice in above-ground tissue of Nipponbare, which may be due to the mechanical wounding upon nematode feeding as found in the rice infested by brown planthopper (Huangfu et al., 2016). At the flowering stage, a sudden burst of *A. besseyi* happened along with the significant downregulation of SA-related genes in Nipponbare, but the significantly upregulation of SA-related genes was observed in Tetep. Further results showed that exogenous application of SA analog, BTH, strongly decreased the rice susceptibility to *A. besseyi*, and corresponding transgenic biosynthesis/signaling defective lines or inhibitor-treated plants showed increased susceptibility. Based on the data provided here, we can conclude that the SA-dependent defense pathway in rice induces a strong defense against the rice foliar nematode *A. besseyi*.

Jasmonates enable plants to defend themselves against attacks by a wide variety of herbivores (Pieterse et al., 2012; Campos et al., 2014), and JA has also been shown to mediate defense against some biotrophic pathogens such as *M. graminicola* and *Xanthomonas oryzae* (Nahar et al., 2011; De Vleesschauwer et al., 2012). Our data showed that exogenous application of MeJA resulted in a strong reduction of nematode number in plants or grains; this observation is in line with previous findings in other nematodes (Nahar et al., 2011, 2012; Khanam et al., 2018). However, previous studies in dicotyledonous plants showed that MeJA application on roots of oat (*Avena sativa*) and spinach (*Spinacia oleracea*) enhanced resistance to the root-knot nematodes due to the stimulated toxic compounds such as 20-hydroxyecdysone and flavone-C-glycosides after MeJA treatment. These compounds are toxic to nematodes *in vitro* and have been implicated in plant defense against plant-parasitic nematodes (Soriano et al., 2004a,b). Our results reveal that JA plays a positive role in rice resistance against *A. besseyi* rather than the toxic effect, which is exemplified by the much higher number of nematodes in JA inhibitor-treated or mutant plants in either seedlings or grains. However, the nematode number in the JA biosynthesis mutant *hebiba* had no significant changes at 25 dai compared with its control Nihonmasari, this probably due to the relatively smaller number of nematodes in the plants at seedling stage.

Generally, the ET pathway synergistically activates JA pathway against necrotrophic pathogens and herbivore insects (Kessler and Baldwin, 2002; Rojo et al., 2003; Howe and Jander, 2008; Pieterse et al., 2012). However, it largely depends on the pathogen lifestyle, infection stage, as well as infected tissues. Our data showed that exogenous application of ethephon leads to a significantly decreased number of nematodes in plants or grains than control, and inhibited ET biosynthesis resulted in an increased susceptibility of the rice plants toward *A. besseyi*. However, mutants with impaired ET signaling (*Ein2b-RNAi*) did not show a significant change in the nematode number neither in plants nor in grains. Considering this result, we deduced that ET biosynthesis, rather than ET signaling, is responsible for ET-mediated systemic defense against *A. besseyi*. Our case is in contrast to the defense pathway of ET involved in rice root innate immunity against *M. graminicola*, in which ET signaling is in charge of ET-mediated systemic defense against *M. graminicola*.

In this study, we presented the responses of two rice cultivars to the foliar nematode *A. besseyi* infection either in nematode number or gene expression. Furthermore, we clarified the roles of SA, JA, and ET pathways in the interaction of rice and *A. besseyi*. Our data show that the resistance in Tetep resulted from the suppression of nematode infection, development, and reproduction. The defense mechanisms in Tetep at least in part were achieved by activating the expression of SA-related defense genes, especially at the flowering stage. Exogenous application of hormones, their analogs, or inhibitors, as well as the analyses of the expression of the marker genes, shows that SA, JA, or ET is involved in rice defense against *A. besseyi*. Nevertheless, the cross talk between the three hormones and other plant hormones in the interaction of rice and *A. besseyi* needs to be further investigated.

## Data Availability Statement

The original contributions presented in the study are included in the article/Supplementary Material, further inquiries can be directed to the corresponding author.

## Author Contributions

HJ and YP initiated and designed the research. JX performed most of the experiments, analyzed the data, and wrote the manuscript. FY and XX helped to conduct the qRT-PCR experiments and analyzed the data. All authors read and approved the final manuscript.

## Conflict of Interest

The authors declare that the research was conducted in the absence of any commercial or financial relationships that could be construed as a potential conflict of interest.

## Publisher’s Note

All claims expressed in this article are solely those of the authors and do not necessarily represent those of their affiliated organizations, or those of the publisher, the editors and the reviewers. Any product that may be evaluated in this article, or claim that may be made by its manufacturer, is not guaranteed or endorsed by the publisher.

## Supplementary Material

Supplementary Figure 1 White tip symptoms observed in Nipponbare and Tetep at the tillering stage. **(A)** The typical white tip symptoms can be observed in Nipponbare. *Left below*: White tip symptoms in detail **(B)**. No typical white tip symptoms in Tetep.

Supplementary Figure 2 Effects of foliar application of plant hormones at high concentrations on rice defense against *Aphelenchoides besseyi* infection. Shoots of six-day-old plants were sprayed until runoff with 250 μM BTH, 500 μM MeJA, and 500 μM ethephon. At 24 h after treatment, plants were inoculated with 250 mixed stages of *A. besseyi*. **(A)** The number of nematodes per plant at 25 dai. Bars represent means ± SE from 3*n* = 45 plants. **(B)** The number of nematodes in 100 mature grains. Bars represent means ± SE from 3*n* = 15 in 100 grains. Different letters indicate statistically significant differences (Tukey test with α = 0.05). MeJA, methyl jasmonate; ethephon (converted to ET in the plant); BTH, benzathiadiazole (SA analog).
