# Supplementary figures and images for: Salicylic Acid, Jasmonate, and Ethylene Contribute to Rice Defense Against White Tip Nematodes Aphelenchoides besseyi
Source: Front Plant Sci. 2022 Jan 20;12:755802. doi: 10.3389/fpls.2021.755802 (PMC8811222; doi:10.3389/fpls.2021.755802)

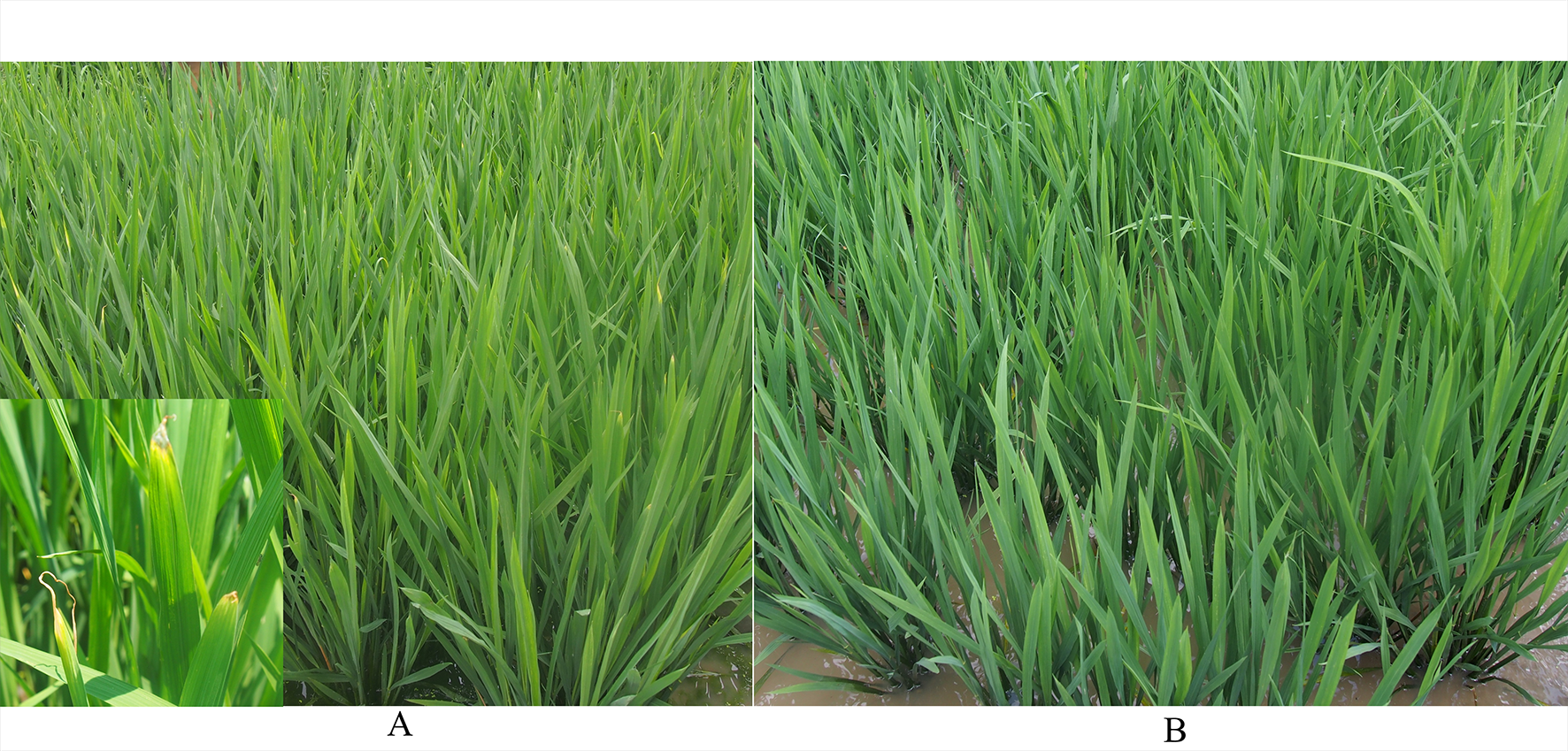

Supplement: Supplementary Figure 1 — White tip symptoms observed in Nipponbare and Tetep at the tillering stage. (A) The typical white tip symptoms can be observed in Nipponbare. Left below: White tip symptoms in detail (B). No typical white tip symptoms in Tetep. [file Image_1.TIF]

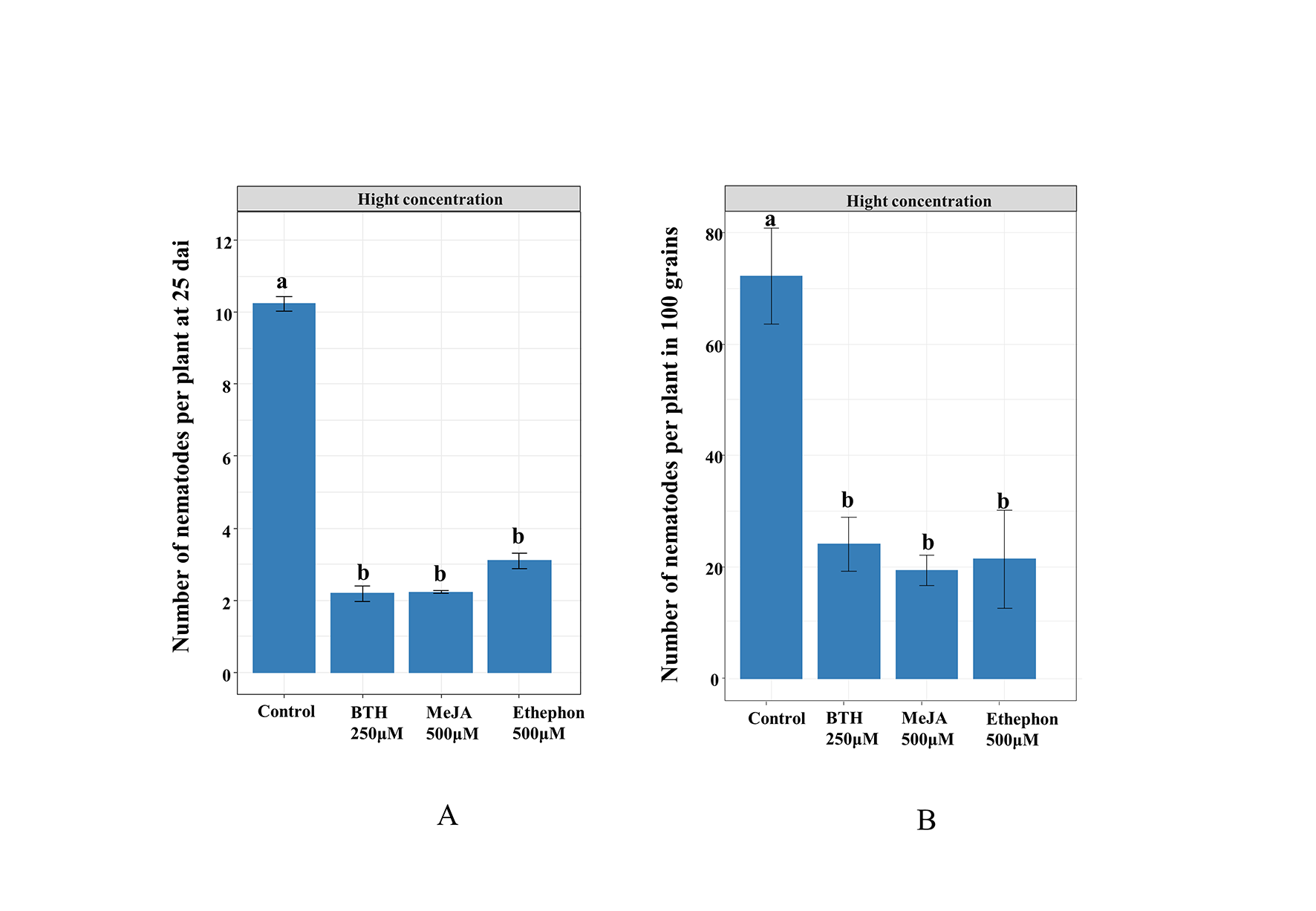

Supplement: Supplementary Figure 2 — Effects of foliar application of plant hormones at high concentrations on rice defense against Aphelenchoides besseyi infection. Shoots of six-day-old plants were sprayed until runoff with 250 μM BTH, 500 μM MeJA, and 500 μM ethephon. At 24 h after treatment, plants were inoculated with 250 mixed stages of A. besseyi. (A) The number of nematodes per plant at 25 dai. Bars represent means ± SE from 3n = 45 plants. (B) The number of nematodes in 100 mature grains. Bars represent means ± SE from 3n = 15 in 100 grains. Different letters indicate statistically significant differences (Tukey test with α = 0.05). MeJA, methyl jasmonate; ethephon (converted to ET in the plant); BTH, benzathiadiazole (SA analog). [file Image_2.tif]
